# Marseilleviruses: An Update in 2021

**DOI:** 10.3389/fmicb.2021.648731

**Published:** 2021-06-02

**Authors:** Dehia Sahmi-Bounsiar, Clara Rolland, Sarah Aherfi, Hadjer Boudjemaa, Anthony Levasseur, Bernard La Scola, Philippe Colson

**Affiliations:** ^1^IHU Méditerranée Infection, Marseille, France; ^2^Institut de Recherche pour le Développement (IRD), Assistance Publique- Hôpitaux de Marseille (AP-HM), MEPHI, Aix-Marseille Université, Marseille, France; ^3^Department of Biology, Faculty of Natural Science and Life, Hassiba Benbouali University of Chlef, Chlef, Algeria

**Keywords:** marseillevirus, *Marseilleviridae*, giant virus, amoeba, *Pimascovirales*, megavirales, human

## Abstract

The family *Marseilleviridae* was the second family of giant viruses that was described in 2013, after the family *Mimiviridae*. *Marseillevirus marseillevirus*, isolated in 2007 by coculture on *Acanthamoeba polyphaga*, is the prototype member of this family. Afterward, the worldwide distribution of marseilleviruses was revealed through their isolation from samples of various types and sources. Thus, 62 were isolated from environmental water, one from soil, one from a dipteran, one from mussels, and two from asymptomatic humans, which led to the description of 67 marseillevirus isolates, including 21 by the IHU Méditerranée Infection in France. Recently, five marseillevirus genomes were assembled from deep sea sediment in Norway. Isolated marseilleviruses have ≈250 nm long icosahedral capsids and 348–404 kilobase long mosaic genomes that encode 386–545 predicted proteins. Comparative genomic analyses indicate that the family *Marseilleviridae* includes five lineages and possesses a pangenome composed of 3,082 clusters of genes. The detection of marseilleviruses in both symptomatic and asymptomatic humans in stool, blood, and lymph nodes, and an up-to-30-day persistence of marseillevirus in rats and mice, raise questions concerning their possible clinical significance that are still under investigation.

## Introduction

Defining any element is based on data and tools that are available at the moment, and definitions can evolve with technological progress (Popper, 2005). The first giant virus was isolated using culturing on *Acanthamoeba* sp. from a water sample collected in 1992 from a cooling tower in Bradford, United Kingdom and identified as a giant virus in 2003. It was initially presumed to be a bacteria and named *Bradford coccus* by T. J. Rowbotham, but it appeared to be a virus, with a particle size of ≈500 nm, named *Acanthamoeba polyphaga Mimivirus* (APMV) (La Scola et al., 2003). This virus was in total opposition to the concept of a virus, defined as a small particle invisible under light microscopy and ultrafilterable through pores with a diameter of 0.2 μm, and with a genetic armamentarium that usually did not exceed a few genes (Lwoff, 1957; La Scola et al., 2003; Raoult et al., 2007; Sharma et al., 2016). This discovery revolutionized the world of virology, sparked the curiosity of many scientists and launched an open debate about the definition and classification of viruses and the universal tree of Life (Raoult et al., 2004; Raoult and Forterre, 2008; Moreira and López-García, 2009; Boyer et al., 2010; Forterre, 2010; Raoult, 2013). Giant virus evolution remains controversial among researchers. Giant viruses of amoeba other than Mimivirus were described (Aherfi et al., 2016b; Colson et al., 2017; Abergel and Claverie, 2020), and they were also related to a monophyletic group of viruses known as nucleocytoplasmic large DNA viruses (NCLDVs) that was described in 2001 and comprises poxviruses, asfaviruses, ascoviruses, iridoviruses, and phycodnaviruses, which have been the subjects of many studies before the discovery of APMV (Wardley et al., 1983; Iyer et al., 2001; Stasiak et al., 2003; Van Etten, 2003; Lefkowitz et al., 2006; Colson et al., 2012; Yutin and Koonin, 2012). We consider giant viruses as having a virion size > 200 nm (Sharma et al., 2016). In 2012, it was proposed to reclassify families of giant amoebal viruses in a new viral order, the Megavirales (Colson et al., 2013a). Since the end of 2019, the International Committee of Taxonomy of Viruses (ICTV) has officially classified giant viruses in the class *Megaviricetes* as part of the phylum *Nucleocytoviricota*, which is part of the kingdom *Bamfordvirae* in the realm *Varidnaviria* (Walker et al., 2019).

Acanthamoeba polyphaga Mimivirus is the first described giant virus and was serendipitously isolated using a coculture strategy on *Acanthamoeba polyphaga* (La Scola et al., 2003). It is the founder of a new viral family officially recognized by the ICTV and named *Mimiviridae* (La Scola et al., 2005a; Suzan-Monti et al., 2006). More than 100 mimivirus strains were thereafter isolated in *Acanthamoeba* spp., and classified in three lineages; A, B and C (Colson et al., 2012).

Marseillevirus (strain T19, later named *Marseillevirus marseillevirus^1^*) was discovered in 2007 as part of the continuation of research on giant amoeba viruses, by culturing on *Acanthamoeba polyphaga*. It is smaller, in terms of particle and genome sizes, than APMV (Boyer et al., 2009). It is the founder of a new viral family, officially recognized and named *Marseilleviridae* (Colson et al., 2013c), which has expanded over the last decade, with more than 50 members isolated; essentially from water, then insects, mussels, but also from humans. These samples were collected from seven countries, over the five continents: Europe, Africa, America, Oceania, and Asia. Marseilleviruses have been classified in the *Megaviricetes* (Walker et al., 2019). They represent a homogeneous group of giant viruses, although they can differ slightly from each other in their genomes and proteomes, the size of their particles, the morphology of the virions and their replicative cycle. After the discovery of mimiviruses, they were the first new giant viruses discovered and preceded numerous others (Colson et al., 2017); among them pandoraviruses (Philippe et al., 2013), pithoviruses (Legendre et al., 2014), faustoviruses (Reteno et al., 2015), molliviruses (Legendre et al., 2015), cedratviruses (Andreani et al., 2016), Pacmanvirus (Andreani et al., 2017), tupanviruses (Abrahão et al., 2018b), and Orpheovirus (Andreani et al., 2018). La Scola et al. also discovered a new type of virus, named virophage, able to parasitize mimiviruses by replicating in their viral factory and integrating in their genome (La Scola et al., 2008; Desnues and Raoult, 2012).

This review presents the most exciting discoveries and developments of the past 10 years concerning marseilleviruses.

## Marseilleviruses: Discovery and History

**FIGURE 1:**
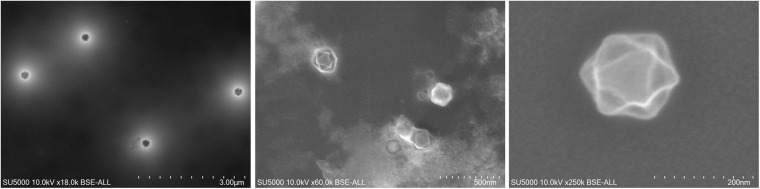
Scanning electron microscopy of marseillevirus T19 particles. Micrographs of viral particles from culture supernatant. Technical settings and scale bars are indicated in the images.

In 2007, Marseillevirus T19 (Figure 1) was isolated in Marseille by a coculture experiment on *Acanthamoeba polyphaga* from a water sample collected in a cooling tower in Paris (Boyer et al., 2009). Cryo-electron microscopy showed a capsid with a diameter of approximately 250 nm, with a shell thickness ≈10 nm separating it from the nucleocapsid by a space ≈5 nm. The marseillevirus surface has fibers of 12 nm in length with globular ends. In 2005, the investigation of a water sample collected in the Seine river around Paris had previously led to the identification of a small intracellular Gimenez-positive coccus (Thomas et al., 2008). In 2011, a second analysis of this sample in a coculture on *Acanthamoeba castellanii* allowed the description of the second giant virus of the family *Marseilleviridae*, named lausannevirus. It has an icosahedral virion with a 190–220 nm diameter without fibrils (Thomas et al., 2011). From there, marseillevirus research in environmental water samples was accelerated, leading from 2013 to 2015 to the isolation of cannes 8 virus from water of a cooling tower in Cannes, southeastern France (Aherfi et al., 2013), and of tunisvirus and saint-charles viruses taken from fresh water collected in fountains in Ariana, a suburb of Tunis, Tunisia and in Marseille, France, respectively (Pagnier et al., 2013; Aherfi et al., 2014). Melbournevirus came from a freshwater pond in Melbourne, Australia (Doutre et al., 2014) and was remarkably similar to marseillevirus and cannes 8 virus. Port-miou virus was isolated from the brackish water flowing from a submarine karstic spring in Port-Miou, Cassis, Marseille, France (Doutre et al., 2015). In 2016, water and soil samples taken from a bank of the Arakawa River, Japan led to the characterization of tokyovirus (Takemura, 2016), and samples of sewage collected in the Pampulha lagoon, Brazil led to the characterization of Brazilian marseillevirus (Dornas et al., 2016). In 2017, noumeavirus (Fabre et al., 2017) and kurlavirus (Chatterjee and Kondabagil, 2017) were isolated from a muddy sample of fresh water collected in a pond near Noumea airport, New Caledonia and a sewage water sample collected in Mumbai, India, respectively. In 2018, two marseillevirus-like viruses were isolated from soil samples collected in an aboriginal village (Serendah village) in Peninsular Malaysia (Tan et al., 2018) and from a fresh water pond bed soil in Shanghai (GenBank MG827395). More recently, in 2019, 15 new members of marseilleviruses, classified in three groups -hokutoviruses, kashiwazakiviruses, and kyotoviruses- were isolated from three water samples from Japan (Aoki et al., 2019). The same year, 15 new marseilleviruses were discovered in sewage, swamp, or wastewater from different locations in Algeria (Supplementary Table 1). Finally, 14 novel marseillevirus isolates have been recently reported from five Japanese aquatic sampling locations, as confirmed by molecular phylogenetic analyses of the major capsid protein (MCP); 11 strains belonged to lineage B, two to lineage A, and one to another lineage close to tokyovirus (Aoki et al., 2021). Therefore, marseilleviruses, like other giant viruses and amoeba, have been shown to be common in water and soil worldwide.

In 2013, the internal organs and digestive tract of a larva from a dipteran, *Eristalis tenax*, were screened. This larva was one of 86 larvae collected from two reservoirs of stagnant water in Tunis, Tunisia, and it allowed isolation of the first giant virus from an insect, which was named insectomime virus (Boughalmi et al., 2013). It was not detected on the surface of the larva, which could indicate the possibility of direct ingestion of the virus or an ingestion of an infected amoeba. Amoeba and non-giant viruses were previously described in insects (Evans and Schwarz, 2011; Otta et al., 2012; Li et al., 2014; Muli et al., 2014).

As marseilleviruses are common in water, golden mussels (Limnoperna fortunei) in southern Brazil were investigated after harvesting them in 2016 from a metal grill that had remained submerged at two meters for 6 months in Guaíba Lake. Decontamination of the surface of the mussels was done, the inner water was collected (dos Santos et al., 2016). Then a coculture procedure of the samples with *Acanthamoeba polyphaga* and *Acanthamoeba castellanii* was performed in order to identify the virus specificity for both species of amoeba. Transmission electron microscopy revealed an icosahedral particle of about 200 nm in diameter named golden marseillevirus (dos Santos et al., 2016). Non-giant viruses and amoeba were also previously described in mollusks, which could therefore be used as an indicator of biological contamination (Mosteo et al., 2016; Ilic et al., 2017).

**FIGURE 2:**
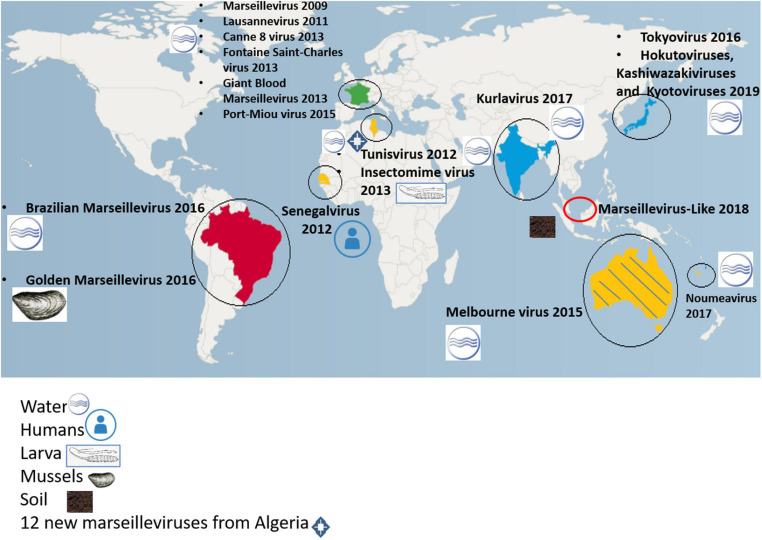
Worldwide distribution of isolated marseilleviruses.

Senegalvirus is the first giant virus isolated from a human, being revealed as one of the components of the gut microbiota. It was isolated from a stool sample which was collected in 2012 in N’Diop, a rural village in Senegal, from a 20-year-old asymptomatic Senegalese man (Lagier et al., 2012) using the coculture strategy on *Acanthamoeba* spp. (Lagier et al., 2012) and metagenomics (Colson et al., 2013b). Electron microscopy revealed the presence of icosahedral particles with a 196 nm diameter (Lagier et al., 2012). Giant blood marseillevirus is the second member of *Marseilleviridae* to be detected in humans, from the blood of a blood donor in 2010 in Marseille, France (Popgeorgiev et al., 2013a). In this study, complete genome sequencing, antigen detection, transmission electron microscopy, fluorescence *in situ* hybridization and cell culture were used and allowed giant blood marseillevirus detection in blood and in inoculated human lymphocytes (Popgeorgiev et al., 2013a). These discoveries suggest that the study of the human virome is still in its infancy (Figure 2).

Currently, metagenomics allows massive investigations of the virome and the microbiome, in addition to studies conducted by co-culture. Genomes from members of the family *Mimiviridae* and other giant virus relatives had already been discovered with this technique (Schulz et al., 2017, 2018). In 2019, for the first time, sequences from five marseillevirus-like viruses were retrieved in deep sea sediment near the Loki’s Castle hydrothermal vent field in Norway (Bäckström et al., 2019).

## Replication Cycle Analysis of Marseilleviruses

### Marseillevirus and Its Different Pathways for Entering Amoeba

**FIGURE 3:**
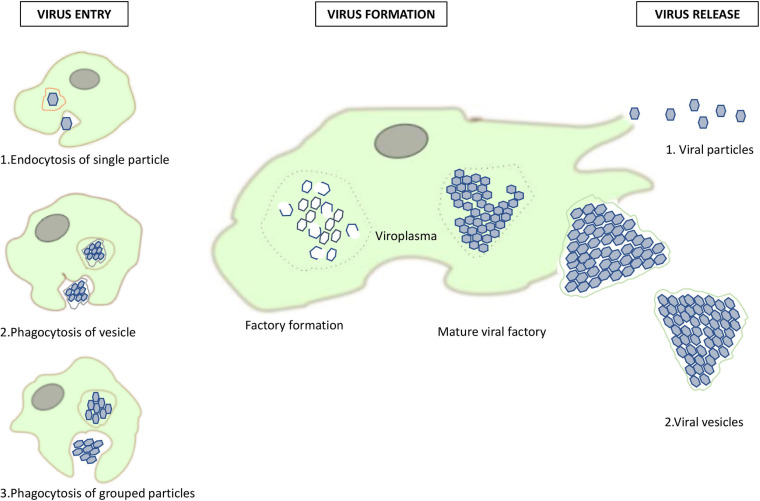
Replication cycle of marseilleviruses. A summary of the different pathways of marseillevirus entry and new virion assembly in amoeba, and their release pathways.

Phagocytosis by *Acanthamoeba* is triggered by particles greater than 500 nm (Weisman and Korn, 1967). It is used by amoebae, and macrophages, to capture mimiviruses (La Scola et al., 2003; Ghigo et al., 2008). The fact that marseilleviruses have a diameter of 250 nm makes this process less likely to occur, although it was described during their discovery (Boyer et al., 2009). In 2016 a study on marseilleviruses and their method for penetrating amoeba was done and revealed different viral strategies for amoeba penetration (Arantes et al., 2016). Scanning and transmission electron microscopy was used to characterized giant vesicles, measuring from 300 nm to more than 1 μm in diameter, depending on the number of viral particles that were encompassed, from dozens to thousands. These vesicles have one or more membranes, likely originating from the endoplasmic reticulum. An interaction of *Acanthamoeba castellanii* with vesicles was reported after 30 min of incubation. When a vesicle has only one membrane, it fuses with the phagosome membrane and releases virus particles inside the amoebal cytoplasm. Alternatively, when vesicles have several membranes, only the external one is merged with the phagosome membrane, keeping vesicles intact inside the amoeba cytoplasm. Another possible way for entry into the amoeba cytoplasm is by phagocytosis of groups of marseillevirus particles, or the endocytosis of single particles (Arantes et al., 2016). Figure 3 summarizes the different pathways of entry of marseilleviruses into amoeba and schematizes the marseillevirus replication cycle.

### Cytopathogenic Effect of Marseilleviruses on Amoeba

During the period from 2007 to 2021 (Rolland et al., 2019), all marseilleviruses were reported to induce the same cytopathogenic effect (CPE) on amoeba; i.e., the rounding of the amoeba and its lysis. Among the 15 marseilleviruses most recently discovered in Japan (Aoki et al., 2019) and classified into 3 groups, two of them: kashiwazakivirus and hokutovirus, belonging to lineage B, presented, in addition to cell rounding, another CPE consisting of aggregation with uninfected cells, which promotes viral dissemination and induces “bunch” formation of amoeba and is original in the family *Marseilleviridae*, but was previously observed with tupanvirus, a mimivirus (Oliveira et al., 2019). Aoki et al. have recently studied the involvement of monosaccharides in bunch formation by amoeba cells infected with a marseillevirus, and they reported that this was observed in lineage B but not lineage A strains. In addition, they observed that galactose inhibited bunch formation whereas mannose and glucose did not.

### Morphogenesis and Release of Virions

All marseilleviruses have been reported to display characteristics for their replication and virion morphogenesis similar to *Marseillevirus marseillevirus*, although with some distinctive features. These steps occur in the cytoplasm in viral factories, which are diffuse areas near the amoeba nucleus. For marseillevirus, encapsidation of viral DNA was described as subsequent to the formation of mature and immature particles (Boyer et al., 2009). The replication cycle was completed within 12 h, with an eclipse phase 2 h post-infection (p.i.) followed by the appearance of a large viral factory in the host cytoplasm at 4 h p.i., by virion morphogenesis inside and around the factory, and by complete lysis of amoeba at 12 h p.i., which released new viral progeny

able to infect other amoeba. Fabre et al. (2017) studied the replication of noumeavirus and reported that it initiates its replication by recruiting the transcription machinery of the amoebal nucleus to their viral factory located in the cytoplasm, while its virions did not incorporate the transcription machinery encoded by their own genes. In fact, it was reported that recruitment of nuclear proteins such as GFP-SUMO from the host nucleus occurred in the early times of the virus replication. These proteins could have essential functions such as the early transcription of mRNA as well as the polyadenylation of viral transcripts. These virus-induced changes, although transient, may allow the viral machinery to take over the host. Giant blood marseillevirus was observed 21 days after inoculation in Jurkat cells, an immortalized line of human T lymphocytes, but did not propagate (Popgeorgiev et al., 2013a). This suggested a possible larger host spectrum for marseilleviruses, not only restricted to amoeba. No virophage, such as those previously described infecting mimiviruses of different lineages (La Scola et al., 2008), has been observed in association with marseilleviruses. It has been hypothesized that the fibrils of mimiviruses were essential for virophage entry (Desnues and Raoult, 2010); also the presence in mimiviruses of protein R135 was associated with fibers (Sobhy et al., 2015). The lack of long fibrils and protein R135 or homologs in marseilleviruses might explain the incapacity of virophages to infect them, but this needs to be further investigated.

## Genomic Study of Marseilleviruses

### General Genomic Characteristics

The genomes of marseilleviruses are double stranded DNA with a size ranging from 348 to 404 kilobases (kb) and with a G + C content between 42.9 and 44.8%. The number of predicted genes varies between 386 for kurlavirus and 491 for Brazilian marseillevirus. A high proportion of these genes corresponds to ORFans (genes without significantly similar sequences in databases) or hypothetical proteins with no function assigned. This is a common feature of giant viruses (Raoult and Boyer, 2010). Several paralogous families of proteins are described in marseillevirus genomes. The most abundant are MORN (Membrane Occupation and Recognition Nexus) repeat-containing proteins, serine and/or threonine kinases (a host-signaling protein family), restriction endonucleases, F-box containing proteins, ankyrin repeat containing proteins, and HNH endonucleases. Interestingly, some particularities were discovered in comparison with other giant viruses such as mimiviruses. Notably, marseilleviruses do not possess elements of translational apparatus. Moreover, three fused genes whose products were identified as homologs of histone-like proteins (doublet-histone H2B/H2A, unknown domain/H2A and archaeal histone/H3) were found in marseilleviruses, which might be involved in viral DNA compaction, protection and/or regulation (Thomas et al., 2011; Erives, 2017).

The high rate of mosaicism observed in *Marseilleviridae* genomes, even in comparison with that of other giant viruses, is singular and intriguing (Boyer et al., 2009). The sympatric life within amoeba for viruses, bacteria, and symbionts facilitates the transfer of genetic material between these microorganisms. This melting pot within amoeba most likely explains the diverse origins of the genes noticed in marseillevirus genomes. In fact, the genes have various putative origins and have been involved in putative exchanges with viruses, essentially giant ones, and bacteria, archaea and eukaryotes, including *Acanthamoeba* spp., the marseillevirus hosts.

A recent study conducted on the noumeavirus DNA confirmed by pulse-field gel electrophoresis and restriction digestion that its genome is circular (Blanca et al., 2020). In addition, these authors conducted a comparative genomic study to analyze the organization of the marseillevirus genomes and reported an asymmetry in sequence conservation along these genomes. Two distinct genomic regions were detected including one that gathers most marseillevirus paralogous genes and underwent genomic rearrangements and another one that is enriched in core genes. In addition, a majority of the genes encoding proteins that compose the viral particles are located in the conserved genomic region. Moreover, the frequency of lately expressed genes was significantly greater in the core region (about two thirds of the genes) than in the other part of the genome (44% of the genes), indicating that genes of the core region are mostly expressed during the late phase of the replication cycle.

A study on promoters performed by Oliveira et al. (2017) showed the abundance of an AT-rich motif (AAATATTT) in multiple copies in intergenic regions of the marseillevirus genome. This motif was associated with 55% of marseillevirus genes, regardless of the lineage. Furthermore, variations in this putative promoter sequence were suspected to alter gene transcription. This investigation suggested that this motif might be an ancient characteristic of the family *Marseilleviridae* due to its abundance in the genomes, and that it might contribute to the genome mosaicism. However, no association of this motif with the temporal transcription levels of marseillevirus genes was observed in a transcriptomic study (Rodrigues et al., 2020).

Some transcripts were retrieved in the capsid of marseillevirus (Boyer et al., 2009). In addition, 49 proteins were identified by proteomics in marseillevirus virions, which comprised the capsid protein, the family B DNA polymerase and also the three histone-like proteins (Boyer et al., 2009). A recent analysis of the marseillevirus transcriptome identified a temporal transcription of genes (Rodrigues et al., 2020). Three categories of transcription profile were detected: early (0, 1, and 2 h p.i.), intermediate (4, 5, 6, and 8 h p.i.) and late expression (10 and 12 h p.i.). Furthermore, an alteration of the host transcription was also observed, with a global decrease in expression of amoeba genes, except for those related to exosome secretion, for which an increase was noted, suggesting the establishment of a defense mechanism by amoeba. It was suspected that the temporal transcription profile with a fast expression of transcription and translation factors was an adaptation of marseillevirus against the defense system developed by the infected amoeba. It is also worthy to note that DNA methylation was reported to be widespread among giant viruses, in two thirds of the giant viral families. Regarding marseilleviruses, some but not all were reported to encode complete sets of components of the restriction-modification (R-M) systems. As a matter of fact, all viruses belonging to lineage A were found to encode a complete R-M system, but this was not the case for members of the other lineages (Jeudy et al., 2020).

### Phylogeny

The family *Marseilleviridae* is classified in five lineages with, as the founding members, marseillevirus (Marseillevirus marseillevirus) in lineage A, and lausannevirus, tunisvirus, Brazilian marseillevirus, and golden marseillevirus in lineages B, C, D, and E, respectively (Boyer et al., 2009; Thomas et al., 2011; Aherfi et al., 2014; Dornas et al., 2016; dos Santos et al., 2016). A phylogenetic tree with most of the marseilleviruses available in the NCBI database was constructed (Figure 5). The MCP gene (the most widely-deposited one) was selected for tree construction with 49 marseilleviruses. The structure of the tree follows the previously-established classification, but the marseillevirus-like genomes generated from metagenomic data form two outgroups relative to the known lineages, one with marseillevirus LCMAC201 and marseillevirus LCMAC202, the second with marseillevirus LCMAC 101, 102, and 103. Their positions between mamavirus used as an outgroup and all lineages of marseilleviruses indicate that they are evolutionarily distant from these latter. A majority of the new marseillevirus-like isolates from IHU Méditerranée Infection are clustered with tunisvirus and insectomime virus in lineage C, whereas the others, including phoenicianvirus, marseillevirus G648, G649, and G650, are clustered with marseilleviruses of lineage A. The 15 marseilleviruses discovered in Japan in 2019 were not yet officially classified. Congruent with Aoki et al. (2019) the seven kyoto virus members are grouped together in lineage A and the two hokutovirus members are grouped in lineage B. Regarding the six kashiwazakivirus members, five are positioned just outside lineage B but are closely related to this lineage, and the fifth one is part of lineage B. These results are congruent with the previous classification of kashiwazakiviruses as a subgroup of lineage B.

**FIGURE 4:**
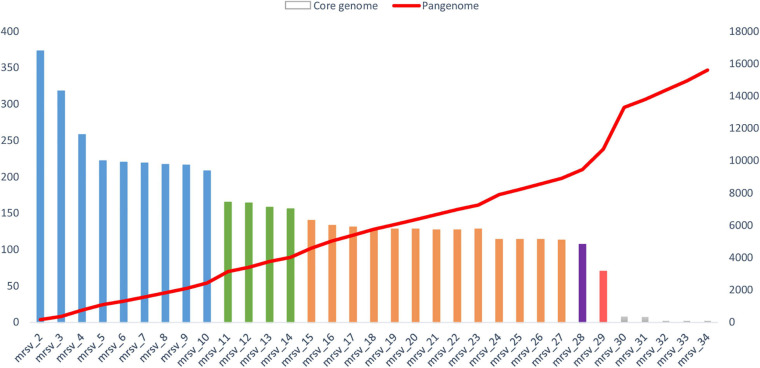
Evolution of the size of the core genome and pangenome of marseilleviruses. The analysis was made by a reciprocal best BLAST hit strategy with significant thresholds for amino acid identity and coverage of 30 and 70%, respectively. The core genome is represented by the histogram column. Each lineage is labeled by a specific color: blue for lineage A, green for lineage B, orange for lineage C, purple for lineage D, and red for lineage E. The pangenome is highlighted by the red curve. The order for inclusion of each marseillevirus in the analysis corresponds to the order shown in Supplementary Table 1, as follows: mrsv_2: Cannes 8 virus; mrsv_3: Melbournevirus; mrsv_4: Tokyovirus A1; mrsv_5: Senegalvirus; mrsv_6: Phoenicianvirus; mrsv_7: Marseillevirus G648; mrsv_8: Marseillevirus G649; mrsv_9: Marseillevirus G650; mrsv_10: Marseillevirus Shangai 1; mrsv_11: Lausannevirus; mrsv_12: Noumeavirus; mrsv_13: Port-miou virus; mrsv_14: Kurlavirus BKC-1; mrsv_15: Tunisvirus; mrsv_16: Insectomime virus; mrsv_17: Marseillevirus N1; mrsv_18: Marseillevirus N16; mrsv_19: Marseillevirus N36; mrsv_20: Marseillevirus N40; mrsv_21: Marseillevirus N50; mrsv_22: Marseillevirus N57; mrsv_23: Marseillevirus N60A; mrsv_24: Marseillevirus N60B; mrsv_25: Marseillevirus NAQ2; mrsv_26: Marseillevirus AM2; mrsv_27: Marseillevirus AM21; mrsv_28: Brazilian marseillevirus; mrsv_29: Golden marseillevirus; mrsv_30: Marseillevirus LCMAC 101; mrsv_31: Marseillevirus LCMAC 102; mrsv_32: Marseillevirus LCMAC 103; mrsv_33: Marseillevirus LCMAC 201; mrsv_34: Marseillevirus LCMAC 202.

**FIGURE 5:**
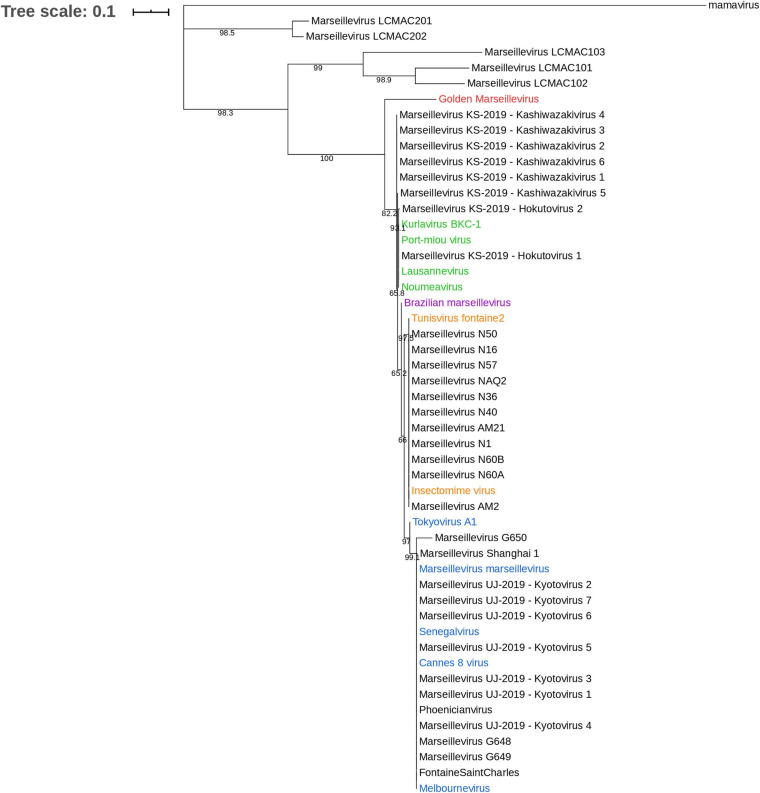
Phylogenetic tree based on the major capsid protein (MCP). The analysis was performed using the Maximum Likelihood method (ML) with a JTT substitution matrix and 1,000 replicates. MCP sequences of marseilleviruses are color-labeled according to their classification into lineages: blue corresponds to lineage A, green to lineage B, orange to lineage C, purple to lineage D, and red to lineage E.

### Pangenome Description

In this review, we updated the core and pangenome of the family *Marseilleviridae*. A total of 34 viruses were included in the analysis, with 15 newly-isolated marseilleviruses discovered at IHU Mediterranée Infection, including one already described in the literature (Schulz et al., 2020), five marseillevirus-like genomes built from metagenomic data (Bäckström et al., 2019), and 14 viruses already characterized belonging to the five *Marseilleviridae* lineages (Supplementary Table 1) (Boyer et al., 2009; Thomas et al., 2011; Aherfi et al., 2013, 2014; Boughalmi et al., 2013; Doutre et al., 2014, 2015; Dornas et al., 2016; dos Santos et al., 2016; Takemura, 2016; Chatterjee and Kondabagil, 2017; Fabre et al., 2017). The study was performed by the addition of the genomes one by one, lineage after lineage. First, we compared the genomes of the marseilleviruses from lineage A, then we added those from lineages B to E until the final inclusion of the five metagenome-derived marseillevirus genomes. The results showed an important difference with or without these latter marseillevirus genomes generated from metagenomic data. We present here the results in two parts, without and with the metagenome-derived genomes.

The total numbers of clusters of genes were 617, 499, and 512 for marseillevirus lineages A, B and C, respectively, and 75, 86, and 88% of these clusters included at least 2 genes for marseillevirus lineages A, B, and C, respectively (Table 1 and Figure 4). All isolated marseillevirus genomes from all known lineages constituted a pangenome encompassing 1,011 clusters of genes, with 29% of them containing one sequence and 71% multiple sequences as determined using the CD-hit software, with thresholds of 30 and 50% for amino acid identity and coverage, respectively (Li and Godzik, 2006). The “strict” core genome (containing at least one sequence of each virus) included 121 clusters corresponding to 4,437 predicted proteins (Supplementary Table 2). It represented 12% of the pangenome. Different classes of proteins were identified in this core genome. The most represented is that of restriction endonucleases and of various other nucleases (including HNH homing endonucleases), with numerous paralogs in each virus. MORN repeat-containing proteins, ankyrin repeat proteins, and serine/threonine kinases also constituted a large number of these proteins. The marseillevirus core genes that are part of the giant virus core genome (Koonin and Yutin, 2010) consisted in 24 clusters of proteins and included the major capsid protein (MCP), the DNA topoisomerase II, the A32-like packaging ATPase, the D5 family helicase-primase, and DNA-directed RNA polymerase subunits. In addition, the clusters comprising the core genome included proteins annotated as putative glycosyltransferases, mannosyltransferase, metallopeptidase, RNA methyltransferase, and ATPase (Supplementary Table 2). The reciprocal best hits analysis performed with the ProteinOrtho software (v6.0.10) with amino acid identity and sequence coverage thresholds of 30 and 70%, respectively (Lechner et al., 2011) reflected the same increasing dynamic of pangenome size, and a total of 70 clusters of proteins included at least one sequence of each of the 29 viruses (0.6% of the full set).

**TABLE 1:** Pangenome and core genome size descriptions for marseilleviruses of lineage A to C and all viruses combined.

	**Lineage A**	**Lineage B**	**Lineage C**	**All lineages**	**Metagenomes**	**All 34 marseilleviruses**
Numbers of clusters (total)	617	499	512	1,011	2,086	3,082
Numbers of clusters with at least two genes	464 (75%)	428 (86%)	453 (88%)	714 (71%)	503 (24%)	1,199 (39%)
Numbers of clusters with one predicted gene	153 (25%)	71 (14%)	59 (12%)	297 (29%)	1,583 (76%)	1,883 (61%)
Strict Core genome	272	319	373	121	3	3

The analysis of the pangenome reinforced the family *Marseilleviridae* classification by lineages. The pangenome size was relatively constant in each lineage (Figure 3). The addition of each lineage one by one coherently increased the pangenome size. Two points of substantial increase appeared between lineage A and B and between lineage A/B/C/D and lineage E. Nevertheless, this latter increase should be nuanced, due to the difference in the number of golden marseillevirus genes deposited in the NCBI database and the number described in the published article [296 versus 483, respectively (dos Santos et al., 2016)].

### Metagenomes

The discovery of marseillevirus sequences in metagenomic data increased the genome size to 763 kb and the range of G + C content extended from 34.2 to 62.3% (Supplementary Table 1). Compared with the other members of the family *Marseilleviridae*, the number of predicted genes in genomes assembled from metagenomes ranged between 427 and a maximum of 793, which is unusual for marseilleviruses, for which the mean number of genes in isolated members is 438. The general picture of the gene composition of genomes assembled from metagenomes is similar to that of genomes from isolated marseilleviruses, with high proportions of ORFans, hypothetical proteins and other previously-described paralogous protein families, as well as a substantial level of mosaicism. In contrast, the presence of aminoacyl-tRNA synthetases and tRNAs were reported in metagenome-derived marseillevirus genomes, whereas they are absent in the genomes of isolated viruses (Bäckström et al., 2019). A total of 15 aminoacyl-tRNA synthetases and 26 tRNA were identified in the five metagenome-derived genomes. This is a brand-new perspective for marseilleviruses that merits confirmation in isolated strains.

The addition of the metagenome-derived marseillevirus genomes in the pangenome analysis dramatically modified the results. The pangenome size indeed reached 3,082 clusters of proteins. Among them, 61% contain a single predicted gene (Table 1). The “strict” core genome was drastically reduced compared to that based on genomes from isolated viruses, with 3 clusters of genes corresponding to the major capsid protein, the A32-like packaging ATPase and the ribonucleoside diphosphate reductase small chain. The extreme reduction of the core genome was also observed by reciprocal best hits analysis (Figure 3).

## Marseilleviruses in Humans

### Marseilleviruses in Asymptomatic Humans

The giant viruses were associated with human pathology with the isolation of a mimivirus from two pneumonia patients, and experimental studies that reported that APMV could cause pneumonia (La Scola et al., 2005b; Saadi et al., 2013; Colson et al., 2016). In addition, the replication of APMV was reported in human macrophages and blood mononuclear cells (Ghigo et al., 2008; Silva et al., 2014), albeit this was not reproducible in the latter cells (Abrahão et al., 2018a) and no viral propagation was reported in these studies. Regarding marseilleviruses, a study of the microbial composition of the gut microbiota found, in the stool of an asymptomatic young Senegalese man, the first marseillevirus, which was named senegalvirus (Lagier et al., 2012). The giant blood marseillevirus was the second to be detected, by metagenomics, in the blood of an asymptomatic blood donor. In addition, PCR, fluorescence *in situ* hybridization (FISH) and electron microscopic analyses performed on Jurkat cells (T lymphocytes) indicated its presence (Popgeorgiev et al., 2013a). A seroprevalence study revealed IgG antibodies to marseillevirus in 13% of 174 blood donors from Marseille and Montpellier, France (Popgeorgiev et al., 2013b). In addition, IgG prevalence for marseillevirus was 23% in 22 thalassemia patients who repeatedly received blood transfusions, and marseillevirus DNA was detected in 9% of these thalassemia patients (Popgeorgiev et al., 2013b). In another study performed on 517 asymptomatic young adults sampled in Lausanne, Switzerland, the seroprevalence for lausannevirus examined by micro-immunofluorescence was 2.5% (Mueller et al., 2013). These results suggest that humans are frequently in contact with marseilleviruses. Moreover, an up-to-30-day persistence of marseillevirus in rats or mice was observed after inoculation through different inoculation routes: intraperitoneal, parenteral, or by aerosolization, and in deep organs (including liver, spleen, lung and nasal-associated lymphoid tissue); no evidence of disease was observed (Aherfi et al., 2018). However, a study of Italian patients showed no evidence of marseillevirus DNA in all 575 blood samples (from 285 healthy donors and 197 immunosuppressed patients) (Macera et al., 2019). Another study also reported the absence of marseillevirus DNA in blood donors and multi-transfused patients (Sauvage et al., 2014). Sequences identified as most related to marseillevirus were detected in metagenomes from asymptomatic people (Rampelli et al., 2016; Bzhalava et al., 2018).

### Marseilleviruses in Symptomatic Humans

Marseilleviruses have also been observed in symptomatic humans. Antigens and DNA of marseillevirus were detected by immunohistochemistry and FISH analyses, respectively, in the lymph node of a febrile 11-month-old child with adenitis of unknown etiology (Popgeorgiev et al., 2013c). This study suggested that marseillevirus infection might become symptomatic in case of an immature or defective immune response causing adenitis. Marseillevirus DNA was also detected in pharyngeal and blood samples from a 20-year-old man with febrile gastroenteritis (Aherfi et al., 2016c). One year later, the patient was still positive to marseillevirus DNA. Detection of marseillevirus with PCR, FISH, direct immunofluorescence, and immunohistochemistry in the lymph node of a 30-year-old woman diagnosed with Hodgkin’s lymphoma has also been reported in association with IgG antibodies to marseillevirus (Aherfi et al., 2016a). The causes of lymph node cancers are not fully understood; links with viruses and bacteria have been reported, with several possible mechanisms of lymphomagenesis such as for Epstein-Barr virus, *Helicobacter pylori*, and *Coxiella burnetii*, which is considered a factor that may promote lymphoid cancer. Previous data, indicating that blood transfusion could be a risk factor for the occurrence of lymphoma, question the possible involvement of marseillevirus in the occurrence of this disease. Therefore, these studies suggest that consideration should be given to marseilleviruses as additional viral causes of human pathology, but other investigations, including those by other teams, should work on this issue to improve our knowledge of the marseillevirus-human relationship. Interestingly, kinases from marseilleviruses were grouped with serine/threonine-protein kinase transforming proteins, and together with kinases from other giant viruses, were reported to display close similarity to viral oncogenes regarding the functional regions and putative substrate binding regions, questioning about a putative role in cancer (Janaki et al., 2020).

## Conclusion

The family *Marseilleviridae* emerged in 2007 and expanded during the past decade to reach more than 50 members in 2021, being the second largest giant viral family after family *Mimiviridae*. During a decade marseilleviruses have been with mimiviruses the only known groups of giant viruses of amoeba. Marseilleviruses have largely contributed, together with mimiviruses and the other giant viruses thereafter discovered, to delineate the remarkable features and define the criteria of what giant viruses are, and to point out how they differ from classical viruses. Although they are among the smallest of these giant viruses, they notwithstanding exhibit several specific phenotypic and genotypic features among giant viruses that warrant particular interest. One of them is for instance the presence of core histone-like genes that form sister clades to all four eukaryotic lineages of core histones. Marseilleviruses have a large worldwide distribution (nine countries, five continents), being found in samples such as water, soil, mussels, insects, and humans. Accordingly, new marseillevirus isolates continue to be described worldwide, including possible members from new lineages (Aoki et al., 2021). Their presence in both symptomatic and asymptomatic patients deserves further investigation.

## Author Contributions

PC, BL, and AL designed and supervised the review. DS-B and CR performed the review of the literature, collected the data, and performed the analyses. HB provided material. DS-B, CR, SA, AL, BL, and PC analyzed the data. DS-B, CR, and PC wrote the manuscript. All authors read the manuscript and approved the final version of the manuscript.

## Conflict of Interest

The authors declare that the research was conducted in the absence of any commercial or financial relationships that could be construed as a potential conflict of interest.

## Footnotes

1 https://talk.ictvonline.org/taxonomy/p/taxonomy-history?taxnode_id=201903801
