# Supplementary material for: Marseilleviruses: An Update in 2021
Source: Front Microbiol. 2021 Jun 2;12:648731. doi: 10.3389/fmicb.2021.648731 (PMC8208085; doi:10.3389/fmicb.2021.648731)
Supplement: Supplementary file 1 [file Data_Sheet_1.docx]

Supplementary Material

# Supplementary Data

**Genome assembly**

The 12 marseillevirus from IHU were assembled using in combination CLC genomics workbench v7.5 (QIAGEN, Aarhus, Denmark) and SPades software with default parameters (Bankevich et al., 2012).

**Genome Annotation**

Gene prediction was computed using Genemarks (Besemer et al., 2005). The predicted proteins with a size less than 50 amino acids were deleted and those between 50 and 99 amino acids analysed with Phyre2 software (Kelley et al., 2015). ORFs having an abnormal tri-dimensional structure were eliminated of the dataset. Predicted genes were annotated using BLASTp request against the non-redundant (nr) protein database with an e-value threshold of 1e-3. Functionnal annotation was refined by using in combination CD-search tool online and InterPro version 77.0. (Marchler-Bauer et al., 2013, Mitchell et al., 2019).

**Core genome – Pangenome Analysis**

Best reciprocal hits were detected by the ProteinOrtho software (v6.0.10) with an amino acid identity percentage and a coverage threshold corresponding to 30 and 70% respectively (Lechner et al., 2011). A clustering of orthologous genes was made using CD-hit software with thresholds of 30 and 50% respectively for amino acid identity and coverage (Li et al., 2006). The core genome and pangenome were estimated by results obtained with CD-hit analyses, refined with ProteinOrtho and classified by lineage.

**Phylogenetic Analysis**

All phylogenetic analyses were performed with the following procedure. A Blastp was computed to find homologs. Then Muscle on MegaX was used for the alignment of sequences (Edgar, 2004). Phylogenetic trees were constructed with IqTree program using the maximum likelihood (ML) framework with Jones-Taylor-Thornton (JTT) as model for amino acid substitution and the ultrafast bootstrap (1000 replicates) (Hoang et al., 2018, Nguyen et al., 2015). Their vizualisation was performed by using iTOL v5 online (Letunic et al., 2016).

**References**

Bankevich, A., Nurk, S., Antipov, D., Gurevich, A.A., Dvorkin, M., Kulikov, A.S., et al. (2012). SPAdes: a new genome assembly algorithm and its applications to single-cell sequencing. *J. Comput. Biol.* 19, 455-477.

Besemer, J., Borodovsky, M. (2005). GeneMark: web software for gene finding in prokaryotes, eukaryotes and viruses. *Nucleic Acids Res.* 33, W451-W454.

Kelley, L.A., Mezulis, S., Yates, C.M., Wass, M.N., Sternberg, M.J. (2015). The Phyre2 web portal for protein modeling, prediction and analysis. *Nat. Protoc.* 10, 845-858.

Marchler-Bauer, A., Zheng, C., Chitsaz, F., Derbyshire, M.K., Geer, L.Y., Geer, R.C., et al. (2013). CDD: conserved domains and protein three-dimensional structure. *Nucleic Acids Res.* 41, D348-D352.

Mitchell, A.L., Attwood, T.K., Babbitt, P.C., Blum, M., Bork, P., Bridge, A., et al. (2019). InterPro in 2019: improving coverage, classification and access to protein sequence annotations. *Nucleic Acids Res.* 47, D351-D360.

Lechner, M., Findeiss, S., Steiner, L., Marz, M., Stadler, P.F., Prohaska, S.J. (2011). Proteinortho: detection of (co-)orthologs in large-scale analysis. *BMC Bioinformatics* 12, 124. doi: 10.1186/1471-2105-12-124., 124-12.

Li, W., Godzik, A. (2006). Cd-hit: a fast program for clustering and comparing large sets of protein or nucleotide sequences. *Bioinformatics* *22,* 1658-1659.

Edgar, R.C. (2004). MUSCLE: a multiple sequence alignment method with reduced time and space complexity. *BMC Bioinformatics* 5, 113.

Nguyen, L.T., Schmidt, H.A., von, H.A., Minh, B.Q. (2015). IQ-TREE: a fast and effective stochastic algorithm for estimating maximum-likelihood phylogenies. *Mol. Biol. Evol.* 32, 268-274.

Hoang, D.T., Chernomor, O., von, H.A., Minh, B.Q., Vinh, L.S. (2018). UFBoot2: Improving the Ultrafast Bootstrap Approximation. *Mol. Biol. Evol.* 35, 518-522.

Letunic, I., Bork, P. (2016). Interactive tree of life (iTOL) v3: an online tool for the display and annotation of phylogenetic and other trees. *Nucleic Acids Res.* 44, W242-W245.

# Supplementary Tables

**Table S1. List of 34 marseilleviruses used for the pangenome analysis with their main characteristics.**

|  | Virus name | Lineage | Genome size (Kilobase pair) | G + C content (%) | Number of predicted genes | Description year | Reference |
| --- | --- | --- | --- | --- | --- | --- | --- |
| 1 | Marseillevirus | A | 368 | 44,7 | 428 | 2009 | [1] |
| 2 | Cannes 8 virus | A | 374 | 44,6 | 483 | 2013 | [2] |
| 3 | Melbournevirus | A | 369 | 44,7 | 403 | 2014 | [3] |
| 4 | Tokyovirus A1 | A | 372 | 44,2 | 470 | 2016 | [4] |
| 5 | Senegalvirus | A | 372 | 44,4 | 419 | 2012 | [5] |
| 6 | Phoenicianvirus | Unclassified | 396 | 44,7 | 433 | 2020 |  |
| 7 | Marseillevirus G648 | Unclassified | 361 | 44,8 | 525 | 2020 |  |
| 8 | Marseillevirus G649 | Unclassified | 372 | 44,7 | 533 | 2020 |  |
| 9 | Marseillevirus G650 | Unclassified | 376 | 44,8 | 545 | 2020 |  |
| 10 | Marseillevirus Shangai 1 | Unclassified | 368 | 44,7 | 472 | 2018 | GenBank MG827395 |
| 11 | Lausannevirus | B | 346 | 42,9 | 444 | 2011 | [6] |
| 12 | Noumeavirus | B | 376 | 42,9 | 452 | 2016 | [7] |
| 13 | Port-miou virus | B | 349 | 42,9 | 410 | 2015 | [8] |
| 14 | Kurlavirus BKC-1 | B | 361 | 42,9 | 386 | 2017 | [9] |
| 15 | Tunisvirus | C | 380 | 43 | 484 | 2014 | [10] |
| 16 | Insectomime virus | C | 386 | 43 | 479 | 2013 | [11] |

|  | Virus name | Lineage | Genome size (Kilobase pair) | G + C content (%) | Number of predicted genes | Description year | Reference |
| --- | --- | --- | --- | --- | --- | --- | --- |
| 17 | Marseillevirus N1 | Unclassified | 404 | 42,8 | 446 | 2020 |  |
| 18 | Marseillevirus N16 | Unclassified | 404 | 42,8 | 447 | 2020 |  |
| 19 | Marseillevirus N36 | Unclassified | 381 | 43 | 426 | 2020 |  |
| 20 | Marseillevirus N40 | Unclassified | 380 | 43 | 426 | 2020 |  |
| 21 | Marseillevirus N50 | Unclassified | 380 | 43 | 425 | 2020 |  |
| 22 | Marseillevirus N57 | Unclassified | 374 | 42,9 | 438 | 2020 |  |
| 23 | Marseillevirus N60A | Unclassified | 380 | 43 | 425 | 2020 |  |
| 24 | Marseillevirus N60B | Unclassified | 380 | 43 | 434 | 2020 |  |
| 25 | Marseillevirus NAQ2 | Unclassified | 402 | 42,9 | 443 | 2020 |  |
| 26 | Marseillevirus AM2 | Unclassified | 404 | 42,9 | 447 | 2020 |  |
| 27 | Marseillevirus AM21 | Unclassified | 384 | 43 | 433 | 2020 |  |
| 28 | Brazilian marseillevirus | D | 362 | 43,3 | 491 | 2016 | [12] |
| 29 | Golden marseillevirus | E | 360 | 43,1 | 296 | 2016 | [13] |
| 30 | Marseillevirus LCMAC 101 | metagenome | 763 | 37,9 | 793 | 2019 | [14] |
| 31 | Marseillevirus LCMAC 102 | metagenome | 395 | 34,2 | 465 | 2019 | [14] |
| 32 | Marseillevirus LCMAC 103 | metagenome | 389 | 62,3 | 427 | 2019 | [14] |
| 33 | Marseillevirus LCMAC 201 | metagenome | 565 | 37,5 | 566 | 2019 | [14] |
| 34 | Marseillevirus LCMAC 202 | metagenome | 705 | 41,6 | 672 | 2019 | [14] |

**Supplementary Table S2. Clusters of proteins of the strict core genome of marseilleviruses**

| **List of all proteins in core genome** | **NCLDV core genes** | **Number of genes in the cluster** |
| --- | --- | --- |
| conserved_restriction_endonuclease |  | 247 |
| hypothetical_protein |  | 206 |
| Vsr/MutH/archaeal_HJR_family_nuclease |  | 67 |
| hypothetical_protein |  | 64 |
| RNA_polymerase_RPB1_large_subunit | X | 61 |
| hypothetical_protein |  | 56 |
| putative_serine/threonine_protein_kinase |  | 56 |
| hypothetical_protein |  | 50 |
| hypothetical_protein |  | 45 |
| hypothetical_protein |  | 44 |
| hypothetical_protein |  | 43 |
| ribonuclease_H |  | 38 |
| MORN_repeat-containing_protein |  | 37 |
| XRN1_5'-3'_exoribonuclease |  | 34 |
| D6/D11-like_helicase | X | 33 |
| DNA_topoisomerase_II | X | 33 |
| HNH_homing_endonuclease |  | 33 |
| serine/threonine_protein_kinase |  | 33 |
| putative_ATPase |  | 32 |
| ankyrin_repeat-containing_protein |  | 32 |
| ATP-dependent_exoDNAse_alpha_subunit |  | 32 |
| ATP-dependent_DNA_ligase | X | 32 |
| major_capsid_protein | X | 31 |
| putative_peptidoglycan_peptidase |  | 31 |
| hypothetical_protein |  | 31 |
| ribonucleoside-diphosphate_reductase_large_chain | X | 31 |
| putative_alkylated_DNA_repair_protein | X | 31 |
| **List of all proteins in core genome** | **NCLDV core genes** | **Number of genes in the cluster** |
| putative_serine/threonine_protein_kinase |  | 31 |
| hypothetical_protein |  | 31 |
| serine/threonine_protein_kinase |  | 31 |
| hypothetical_protein |  | 31 |
| hypothetical_protein |  | 31 |
| serine/threonine_protein_kinase |  | 31 |
| translation_elongation_factor_EF-1alpha | X | 31 |
| conserved_putative_membrane_protein |  | 31 |
| putative_mannosyltransferase |  | 31 |
| VLTF2 | X | 31 |
| dihydrofolate_reductase-thymidylate_synthase | X | 30 |
| putative_glycosyltransferase |  | 30 |
| 2H-phosphodiest_domain-containing_protein | X | 30 |
| hypothetical_protein |  | 30 |
| putative_A32-like_packaging_ATPase | X | 30 |
| hypothetical_protein |  | 30 |
| transcription_initiation_factor_TFIIB | X | 30 |
| hypothetical_protein |  | 30 |
| hypothetical_protein |  | 30 |
| Nudix_hydrolase | X | 30 |
| putative_Flap_endonuclease | X | 30 |
| ubiquitin-like_protein |  | 30 |
| serine/threonine_protein_kinase |  | 30 |
| putative_secreted_protein |  | 30 |
| hypothetical_protein |  | 30 |
| hypothetical_protein |  | 30 |
| hypothetical_protein |  | 30 |
| D5_family_helicase-primase | X | 30 |
| hypothetical_protein |  | 30 |
| **List of all proteins in core genome** | **NCLDV core genes** | **Number of genes in the cluster** |
| hypothetical_protein |  | 30 |
| ribonucleoside-diphosphate_reductase_small_subunit | X | 30 |
| AAA-family_ATPase |  | 30 |
| hypothetical_protein |  | 30 |
| putative_membrane_protein |  | 30 |
| putative_nuclease |  | 30 |
| inactivated_thioredoxin/glutaredoxin |  | 30 |
| putative_thioredoxin |  | 30 |
| papain-like_cysteine_peptidase |  | 30 |
| putative_NUDIX_hydrolase | X | 30 |
| hypothetical_protein |  | 30 |
| hypothetical_protein |  | 30 |
| hypothetical_protein |  | 30 |
| mRNA_capping_enzyme | X | 30 |
| serine/threonine_protein_kinase |  | 30 |
| histone_H2A_domain-containing_protein |  | 30 |
| bacterial_MORN_repeat-containing_protein |  | 30 |
| hypothetical_protein |  | 30 |
| putative_ribonuclease_III |  | 30 |
| putative_membrane_protein |  | 30 |
| hypothetical_protein |  | 30 |
| membrane_protein |  | 30 |
| zinc_finger_protein |  | 30 |
| metal-dependent_phosphohydrolase |  | 30 |
| superfamily_II_helicase | X | 30 |
| serine/threonine_protein_kinase |  | 30 |
| hypothetical_protein |  | 30 |
| putative_endonuclease_4 |  | 30 |
| putative_nuclease |  | 30 |
| hypothetical_protein |  | 30 |
| **List of all proteins in core genome** | **NCLDV core genes** | **Number of genes in thecluster** |
| hypothetical_protein |  | 30 |
| thymidine_kinase |  | 30 |
| hypothetical_protein |  | 30 |
| ankyrin_repeat-containing_protein |  | 30 |
| hypothetical_protein |  | 30 |
| hypothetical_protein |  | 30 |
| DNA-directed_RNA_polymerase_subunit_2 | X | 30 |
| putative_RNA_methyltransferase |  | 30 |
| DNA-directed_RNA_polymerase_subunit_RPB5 | X | 30 |
| Rossmann-fold_nucleotide-binding_protein |  | 30 |
| class_3_lipase |  | 30 |
| papain-like_cysteine_peptidase |  | 30 |
| disulfide_oxidoreductase |  | 30 |
| hypothetical_protein |  | 30 |
| hypothetical_protein |  | 30 |
| hypothetical_protein |  | 30 |
| ankyrin_repeat-containing_protein |  | 30 |
| zinc_finger/DNA-binding_protein |  | 30 |
| hypothetical_protein |  | 30 |
| D6/D11-like_helicase | X | 30 |
| hypothetical_protein |  | 30 |
| SWIB/MDM2_domain-containing_protein |  | 30 |
| hypothetical_protein |  | 30 |
| putative_DNA_repair_exonuclease |  | 30 |
| WD_repeat-containing_protein |  | 30 |
| hypothetical_protein |  | 30 |
| putative_glycosyltransferase |  | 30 |
| hypothetical_protein |  | 30 |
| putative_RuvC-like_Holliday_junction_resolvase | X | 30 |
| **List of all proteins in core genome** | **NCLDV core genes** | **Number of genes in thecluster** |
| hypothetical_protein |  | 29 |
| hypothetical_protein |  | 29 |
| hypothetical_protein |  | 29 |
| putative_metallopeptidase_WLM |  | 29 |
| hypothetical protein |  | 29 |
| hypothetical_protein |  | 29 |
